# Highly active RRMS and ocrelizumab after failure of alemtuzumab therapy

**DOI:** 10.1186/s12883-020-01789-y

**Published:** 2020-05-21

**Authors:** Martin Vališ, Pavel Ryška, Simona Halúsková, Blanka Klímová, Zbyšek Pavelek

**Affiliations:** 1 Department of Neurology, Faculty of Medicine and University Hospital Hradec Králové, Charles University in Prague, 581, 500 05 Hradec Králové, Czech Republic; 2 Department of Radiology, University Hospital Hradec Králové, Hradec Králové, Czech Republic

**Keywords:** Multiple sclerosis, Case report, Relapse, Disease progression, Alemtuzumab, Treatment failure, Lymphocytes, Ocrelizumab

## Abstract

**Background:**

A high multiple sclerosis activity while on alemtuzumab is rather uncommon compared to moderate-efficacy drugs. The purpose of this case report is to present a case of a 37-year-old female patient with bronchial asthma and no other medical history, whose disease activity required switching from dimethyl fumarate to fingolimod, then to alemtuzumab and finally to ocrelizumab.

**Case presentation:**

In our patient, two severe attacks were observed and treated after administration of the first pulse of alemtuzumab. After six months of therapy, patient’s immunological profile showed the expected decrease in CD4+ and CD8+ T-cells and, markedly increased values of CD19+ B-cells. Surprisingly memory B-cells, which typically repopulate very slowly following alemtuzumab treatment, were above baseline levels. Regular administration of ocrelizumab based on a standardised scheme, after the alemtuzumab therapy failure, resulted in the stabilisation of the patient’s condition both clinically and radiologically.

**Conclusion:**

Thus, when the alemtuzumab treatment is unsuccessful, the authors recommend testing T- and B-cell levels and proceeding with an early switch to ocrelizumab if high B-cell counts are found.

## Background

Multiple sclerosis (MS) is a chronic inflammatory demyelinating disease of the central nervous system, characterised by a very broad heterogeneity in terms of clinical features, genetics, pathogenesis and responsiveness to treatments. Treatment of MS includes symptomatic therapies, management of acute relapses (corticosteroids or plasmapheresis) and therapy with disease-modifying drugs (DMDs) which reduces relapse rate, delays accumulation of disability and has beneficial effects on magnetic resonance imaging measures affecting the volume of lesions, number of active lesions, and slowing down the progression of brain atrophy. As confirmed by research and clinical trial results, early diagnosis and treatment in the initial stages of MS can significantly slow disease progression, preserve performance status in the long run, and prevent permanent damage to nerve structures [1]. Therapy of a clinically isolated syndrome and relapsing-remitting forms of MS is usually commenced using the first-line drugs, that include injections of glatiramer acetate (GA), interferons (beta-1a and beta-1b), and oral dimethyl fumarate and teriflunomide. If the response is inadequate or the treatment is poorly tolerated, the patient is switched to another drug within the same line or the therapy is escalated by utilizing intravenous natalizumab, alemtuzumab, ocrelizumab, or oral fingolimod or cladribine.

## Case presentation

**Fig. 1:**
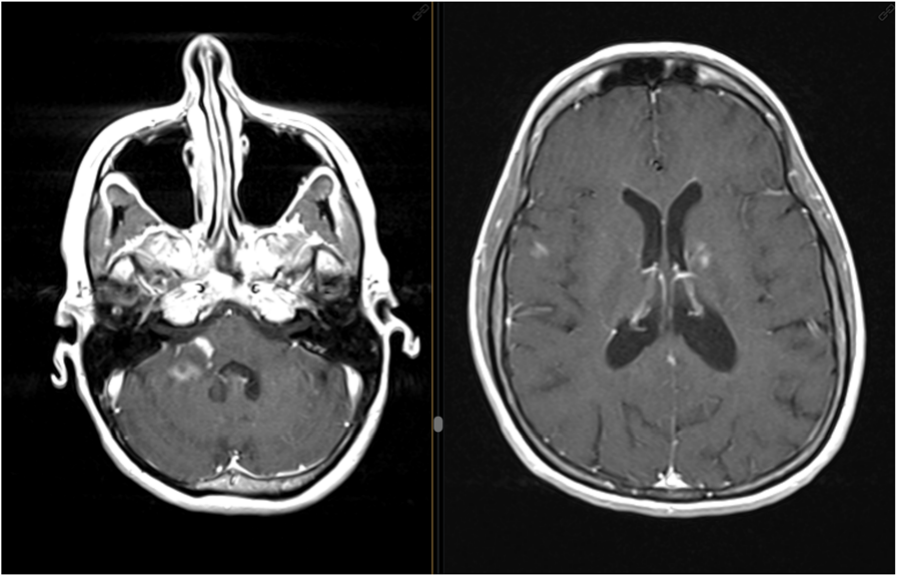
T1 Gd sequences

A 37-year-old female was diagnosed with relapsing-remitting MS (RRMS) in 2005 after two episodes of left optic neuritis. Her baseline Expanded Disability Status Scale (EDSS) was 2.0 and DMD therapy was initiated with glatiramer acetate (GA) 20 mg/daily. In 2014 GA was switched to dimethyl fumarate (DMF) due to persistent local injection site reactions. Patient experienced relapse of left sensory hemisyndrome in February 2017 and protracted attack of quadrupyramidal syndrome requiring repeated intravenous methylprednisolone (IVMP) infusions, with EDSS progression to 4.0 in April 2017. Consequently, the treatment was escalated to fingolimod in June 2017, considering John Cunningham virus (JCV) seropositivity (fingolimod and natalizumab were available at that time). A severe brainstem attack was treated in May 2018 by high-dose IVMP and series of plasma exchange. EDSS worsened to 4.5 and performed magnetic resonance imaging (MRI) revealed noticeable disease progression (Fig. 1).

**Fig. 2:**
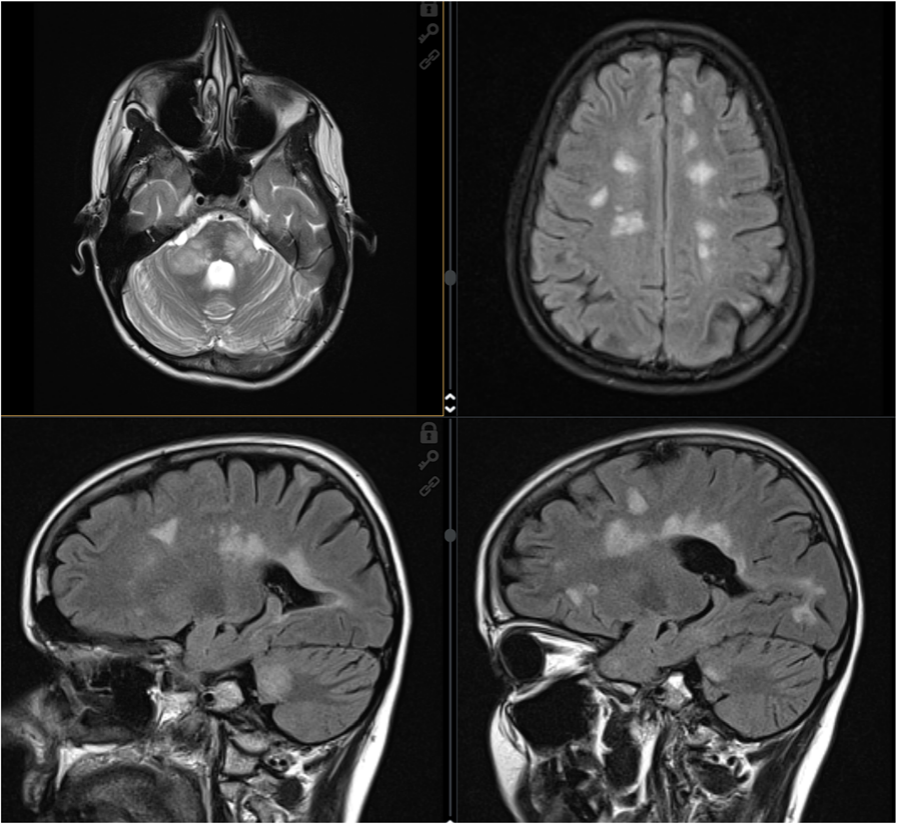
4/2019 – T2 and FLAIR images. Multiple MS lesions of supratentorial and infratentorial white matter

Given the failure of fingolimod therapy and the persistent presence of positive anti-JCV antibodies (3.22), treatment with alemtuzumab was initiated in October 2018. Although the disease course was favorable during the next 4 months and EDSS decreased to 3.5, patient had rather serious brainstem relapse in February 2019, with EDSS progression to 5.5. Follow-up MRI displayed seven new lesions, one with post-contrast opacification. Another attack clinically presented by central right hemiparesis and expressive aphasia was treated in April 2019, with additional EDSS worsening to 6.5. MRI showed further disease progression in terms of enlarged MS lesions as well as activity of multiple new plaques, both supra- and infratentorial (Fig. 2).

**Fig. 3:**
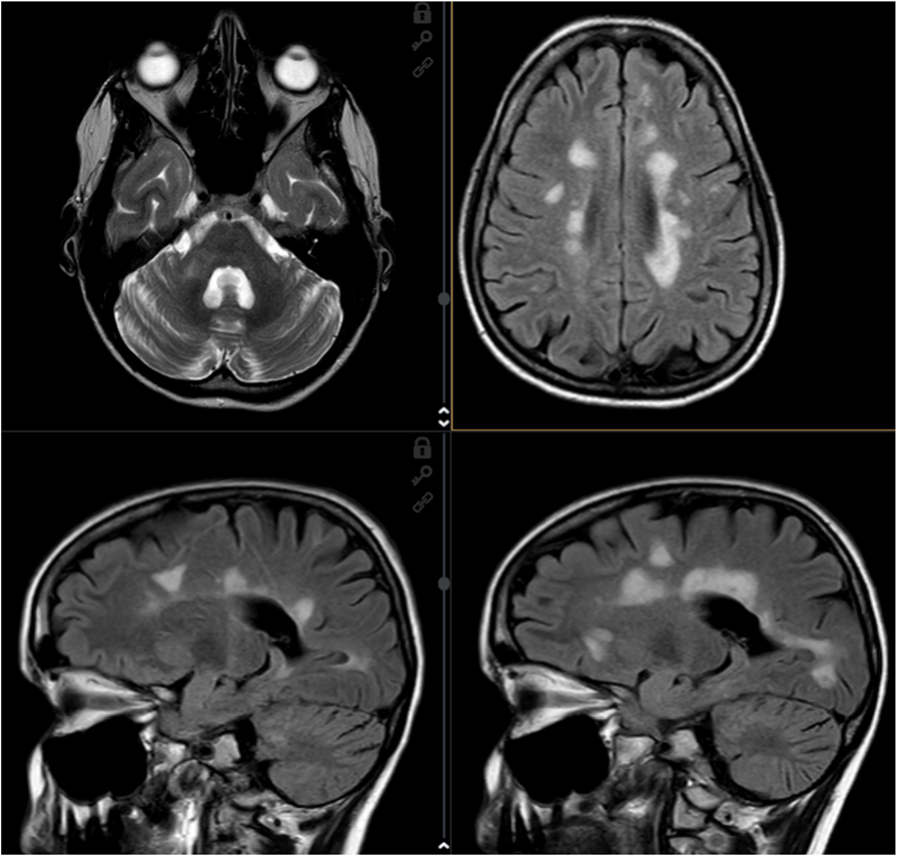
12/2019 T2 and FLAIR images. MRI after switch to ocrelizumab. Almost complete regression of inratentorial RS lesions. Supratentorial lesions without progression

Given the insufficient response to alemtuzumab and ongoing disease activity, the patient was switched to ocrelizumab in June 2019. The therapy has been beneficial so far and patient remains clinico-radiologically stable, with no other attacks, EDSS has stabilized at 4.5, and the latest MRI scan from December 2019 demonstrated significant improvement of the finding (Fig. 3).

## Discussion and conclusion

The present case report describes the 15-year period of a female patient with aggressive RRMS who received GA, DMF, fingolimod, alemtuzumab and ocrelizumab, respectively. Switching from DMF to fingolimod and from fingolimod to alemtuzumab is not unusual in common clinical practice. However, high MS activity while on alemtuzumab is rather uncommon compared to moderate-efficacy drugs. Alemtuzumab depletes circulating T- and B-lymphocytes with the lowest cell counts observed at the first post-baseline assessment. B-cell counts normalise within 3 months of administration while T-cell counts recover over a longer period of time [2]. After 6 months of therapy, patient’s immunological profile showed the expected decrease in CD4+ and CD8+ T-cells and, markedly increased values of CD19+ B-cells (3.4 times the upper reference limit of the laboratory). As expected, an increase in naïve B-lymphocytes was recorded (4.3 times the upper limit of the laboratory). However, high values of class-switched memory B-cells (3.5 times the upper limit of the laboratory) were detected. These results are unexpected when compared to the results of Baker et al. [3], where after 6 months of treatment absolute CD19+ B lymphocytes were + 5% vs. baseline and memory B-cells – 80% vs. baseline. Previous works demonstrated that memory B-cells typically repopulate very slowly [3–5] and according to Akgün et al. [5] this process took years. Therefore, it is of interest that depletion of cells within this subset and controlling their repopulation have been associated with treatment efficacy [4, 6]. Thus, the treatment was switched to ocrelizumab, which selectively targets the CD20-positive B-lymphocytes resulting in B-cell depletion whereas natural immunity and total T-cell count remain unaffected [7, 8]. Whilst, a lack of CD4+ T-cell decrease in alemtuzumab-treated patients, or their rapid repopulation, has been suggested to lead to persistent relapses [5, 9, 10], this has remains controversial [11, 12]. Nevertheless, this study is consistent with disease breakthrough relating to B-cell activity [6]. Therefore, we recommend testing B-lymphocytes in case of failure of alemtuzumab and considering early change of treatment to ocrelizumab.

## Data Availability

On request from the head of the Department of Neurology of the University Hospital of Hradec Kralove.

## Abbreviations

DMF: dimethyl fumarate
DMDs: disease-modifying drugs
EDSS: Expanded Disability Status Scale
GA: glatiramer acetate
IVMP: intravenous methylprednisolone
JCV: John Cunningham virus
MRI: magnetic resonance imaging
MS: multiple sclerosis
RRMS: relapsing-remitting multiple sclerosis
